# Novel genetically engineered mouse models for clear cell renal cell carcinoma

**DOI:** 10.1038/s41598-023-35106-7

**Published:** 2023-05-22

**Authors:** Johannes C. van der Mijn, Kristian B. Laursen, Leiping Fu, Francesca Khani, Lukas E. Dow, Dawid G. Nowak, Qiuying Chen, Steven S. Gross, David M. Nanus, Lorraine J. Gudas

**Affiliations:** 1 Department of Pharmacology, New York Presbyterian Hospital, Weill Cornell Medicine, 1300 York Ave, New York, NY 10065 USA; 2 Division of Hematology/Oncology, Department of Medicine, New York Presbyterian Hospital, Weill Cornell Medicine, New York, NY USA; 3 Department of Biochemistry, New York Presbyterian Hospital, Weill Cornell Medicine, New York, NY USA; 4 Department of Pathology, New York Presbyterian Hospital, Weill Cornell Medicine, New York, NY USA; 5 Department of Urology, New York Presbyterian Hospital, Weill Cornell Medicine, New York, NY USA; 6 Meyer Cancer Center, New York, USA; 7 Present Address: Department of Medical Oncology, The Netherlands Cancer Institute, Amsterdam, The Netherlands; 8 Present Address: Paratus Sciences, Alexandria Bld. West, New York, USA

**Keywords:** Biological techniques, Cancer, Molecular biology, Oncology

## Abstract

Genetically engineered mouse models (GEMMs) are important immunocompetent models for research into the roles of individual genes in cancer and the development of novel therapies. Here we use inducible CRISPR-Cas9 systems to develop two GEMMs which aim to model the extensive chromosome p3 deletion frequently observed in clear cell renal cell carcinoma (ccRCC). We cloned paired guide RNAs targeting early exons of *Bap1*, *Pbrm1*, and *Setd2* in a construct containing a Cas9^D10A^ (nickase, hSpCsn1n) driven by tetracycline (tet)-responsive elements (TRE3G) to develop our first GEMM. The founder mouse was crossed with two previously established transgenic lines, one carrying the tet-transactivator (tTA, Tet-Off) and one with a triple-mutant stabilized HIF1A-M3 (TRAnsgenic Cancer of the Kidney, TRACK), both driven by a truncated, proximal tubule-specific *γ-glutamyltransferase 1* (ggt or γGT) promoter, to create triple-transgenic animals. Our results indicate that this model (BPS-TA) induces low numbers of somatic mutations in Bap1 and Pbrm1 (but not in Setd2), known tumor suppressor genes in human ccRCC. These mutations, largely restricted to kidneys and testis, induced no detectable tissue transformation in a cohort of 13 month old mice (N = 10). To gain insights into the low frequencies of insertions and deletions (indels) in BPS-TA mice we analyzed wild type (WT, N = 7) and BPS-TA (N = 4) kidneys by RNAseq. This showed activation of both DNA damage and immune response, suggesting activation of tumor suppressive mechanisms in response to genome editing. We then modified our approach by generating a second model in which a ggt-driven, cre-regulated Cas9^WT^(hSpCsn1) was employed to introduce *Bap1*, *Pbrm1*, and *Setd2* genome edits in the TRACK line (BPS-Cre). The BPS-TA and BPS-Cre lines are both tightly controlled in a spatiotemporal manner with doxycycline (dox) and tamoxifen (tam), respectively. In addition, whereas the BPS-TA line relies on paired guide RNAs (gRNAs), the BPS-Cre line requires only single gRNAs for gene perturbation. In the BPS-Cre we identified increased Pbrm1 gene-editing frequencies compared to the BPS-TA model. Whereas we did not detect Setd2 edits in the BPS-TA kidneys, we found extensive editing of Setd2 in the BPS-Cre model. Bap1 editing efficiencies were comparable between the two models. Although no gross malignancies were observed in our study, this is the first reported GEMM which models the extensive chromosome 3p deletion frequently observed in kidney cancer patients. Further studies are required (1) to model more extensive 3p deletions, e.g. impacting additional genes, and (2) to increase the cellular resolution, e.g. by employing single-cell RNAseq to ascertain the effects of specific combinatorial gene inactivation.

## Introduction

Clear cell renal cell carcinoma (ccRCC) is the most common form of primary kidney cancer in adults. A combination of multi-region tumor sampling and whole-genome sequencing has identified large somatic copy number losses of chromosome 3p, probably occurring in childhood or adolescence through chromothripsis, a disease initiating event^1–3^. The genes on chromosome 3p include VHL, PBRM1, BAP1, and SETD2, and whereas single gene inactivation has been modelled in GEMMs, the cumulative effects of simultaneous loss of several of these genes have not been assessed. These genes are neighbors on human chromosome 3p, but in mice the genes are located on different chromosomes (*Vhl;* chr6, *Bap1*; chr14, *Setd2*; chr9, *Pbrm1*; chr14), and consequently the simultaneous loss of function is challenging to achieve. While the inactivation of *VHL* is considered the rate-limiting event in carcinogenesis, the majority of ccRCC tumors also contain deletions of the genomic loci of *PBRM1*, *SETD2*, and/or *BAP1*^2,3^. In many patients these deletions are present in the trunk of the phylogenetic tree, which indicates that they likely contribute to early clonal expansion^4^. Particularly, the accumulation of multiple driver events in early ancestral clones was found to be associated with rapid disease progression and poor overall survival^4^.

The combination of VEGF- and immune checkpoint-inhibitors recently emerged as novel treatment strategy for patients with metastatic ccRCC^5–7^. Although these treatment strategies represent an improvement in care, few patients with metastatic ccRCC are being cured with the current therapies and treatment resistance invariably occurs. GEMMs have been developed to enhance our understanding of drug resistance mechanisms and facilitate the development of novel therapeutic agents. Previous research showed that conditional inactivation of *Vhl* in the kidneys of mice induces the development of premalignant lesions^8^. Subsequent research has demonstrated that the formation of these lesions is primarily mediated by *Hif1α*^9–12^. Consistently, kidneys expressing a constitutive Hif1α protein display numerous features of early stage ccRCC^13,14^. Additional studies, which used the Cre/lox site-specific recombination system, revealed that additional genomic alterations are required to induce renal cancers. For example, combined deletion of *Vhl* and *Pbrm1* or *Bap1* resulted in multifocal, transplantable kidney cancers arising from the proximal tubules of mice^9,10^. These models are, however, limited by the formation of bilateral renal cysts and early induction of kidney failure and do not progress to metastatic disease. Others generated metastatic ccRCC models through expression of a *Myc* transgene, in combination with *Vhl* and *Cdkn2a* deletion, or through a combined deletion of *Vhl*, *Trp53,* and *Rb1*^15,16^. While these GEMMs are valuable tools for preclinical research, they are at least partly driven by mutations that do not typically occur in the early stages of human ccRCC development^2,3^. Hence novel GEMMs of metastatic ccRCC are needed to increase our understanding of disease progression towards metastatic disease and to aid in the development of novel therapies.

Significant improvements have been made in the development of GEMMs in the past decade by the introduction of novel gene editing strategies. Inducible CRISPR-Cas9 vectors have been developed that provide temporal and spatial control of the induction of genetic alterations in mice^17^. We previously developed the TRACK (TRAnsgenic Cancer of the Kidney), model, which contains *HIF1A-M3*, a transgene with three mutations (P402A, P564A, N803A) in the oxygen-dependent degradation domain making the HIF1A-M3 protein resistant to degradation by pVHL^13^. The expression of this constitutively active transgene is driven by the ggt-promoter, which is highly active in proximal tubule cells of the kidney. While the kidneys in this model display numerous features of early stage ccRCC^13,14^, TRACK mice don’t form invasive or metastatic ccRCCs. Aiming to develop novel GEMMs for ccRCC, we introduced into the TRACK model an allele for somatic gene inactivation of *Bap1, Pbrm1*, and *Setd2* in a stochastic combinatorial manner.

Specifically, we cloned six paired guide RNAs (gRNAs) targeting *Bap1*, *Pbrm1,* and *Setd2* in a vector containing humanized Cas9^D10A^ driven by a promoter containing tetracycline-responsive elements (TRE3G). Subsequently, we performed targeted integration into embryonic stem cells (ESCs) and injected these KH2 ESCs into mouse blastocysts for tetraploid complementation. The BPS/R26/rtTA founder mice were crossed with doubly transgenic mice derived from previously established mouse lines; the TRACK mice carrying the triple-mutant stabilized *HIF1A-M3* allele, and the tet-TA line in which the tet-transactivator (tTA, Tet-Off) is driven by the proximal tubule-specific *γ-glutamyltransferase 1* (ggt) promoter. This generated the BPS-TA triple transgenic mouse model, in which the highly specific Cas9^D10A^ (nickase) is expressed in proximal tubule cells of the kidneys in a dox dependent manner^13,18^. Next, we crossed mice with an established mouse line expressing the CreER recombinase under the control of a proximal tubule-specific, truncated *γ-glutamyltransferase 1* (ggt) promoter to generate the second BPS-Cre quadruple transgenic model in which Cre-mediated excision of the LSL (lox-stop-lox) element, activates continuous expression of Cas9^WT^ specifically in proximal tubule kidney cells. In this second BPS-Cre GEMM somatic mutations arise from activity of a more potent, but less target-site specific wild type Cas9 endonuclease, which is active after a single tamoxifen injection. Our results demonstrate that somatic mutations in all three tumor suppressor genes can be induced selectively in the kidney proximal tubules using these two models, BPS-TA and BPS-Cre.

## Materials and methods

### Construction of c3GIC9n-BPS for targeting of Bap1, Pbrm1, and Setd2

Six paired single guide RNAs (gRNAs) for targeting of the mouse Bap1, Pbrm1, and Setd2 genes were each cloned into the BbsI site of the pX461 plasmid, Addgene #48,140 (Table 1). The six U6 gRNA cassettes were amplified by PCR and NsiI/SbfI-cut PCR fragments (390 bp) were cloned sequentially into NsiI-opened, dephosphorylated targeting construct c3GIC9n, Addgene #62,192^17^. See Table 1 for details. The integrities of each of the six gRNA expression cassettes were confirmed by Sanger sequencing. In addition to the gRNA expression cassettes, the resulting c3GIC9n-BPS construct contains a GFP-IRES-Cas9^D10A^ expression cassette, coding for enhanced green florescent protein (GFP) and a HA-tagged version of the humanized *S. Pyogenes* Cas9^D10A^ (or hSpCsn1n) with double-nicking requirement for the induction of double strand breaks with high fidelity ^19^. Expression of the GFP-IRES-Cas9^D10A^ cassette is regulated by the 3rd generation tetracycline-regulated promoter (TRE3G).

**Table 1:** Primers for construction of gRNA cassettes.

Primer	Sense	Anti-sense
Bap1-F	caccGCTCTTCGATCCATTTGAACAGG	aaacCCTGTTCAAATGGATCGAAGAGC
Bap1-R	caccgCCGCCGCAAGGTTTCTACGTTGG	aaacCCAACGTAGAAACCTTGCGGCGGC
Pbrm1-F	caccgTTCATCCTTATAGTCTCGGATGG	aaacCCATCCGAGACTATAAGGATGAAC
Pbrm1-R	caccgTGTTCATTAGGGCTCCAAAGCGG	aaacCCGCTTTGGAGCCCTAATGAACAC
Setd2-F	caccgAACTTTTGTCTTCGTGCCTTTGG	aaacCCAAAGGCACGAAGACAAAAGTTC
Setd2-R	caccGGAGGAACAGGGACGGCAAAAGG	aaacCCTTTTGCCGTCCCTGTTCCTCC
U6-NsiI	ATGCTATGCATGAGGGCCTATTTCCCATGATT	
U6-SbfI	TGACACCTGCAGGTCTAGCTCTAAAACAAAAAAGC	

### Detection of Bap1, Pbrm1, and Setd2 genome editing

Transgenic mice were sacrificed (or ESCs were harvested) and genomic DNA was extracted from various tissues using the QIAamp DNA micro Kit (Qiagen #56,304). Genomic target regions were amplified by Phusion PCR (Thermo, #F530L) using primers spanning the Bap1, Pbrm1, and Setd2 targeted regions. See Table 2 for details. The PCR products were column purified, hybridized, and subjected to a series of melt-anneal temperature cycles, the analyzed by Surveyor/T7E1 assay in which the heteroduplexes were selectively digested with a DNA T7E1 endonuclease. The PCR products were subsequently visualized on a 2% agarose gel. For an independent confirmation retarded migration of the heteroduplexed PCR products was assayed by polyacrylamide gel (7.5%) electrophoresis, after which the PCR products were visualized by ethidium bromide staining^20^. The PCR products from BPS-TA mice were analyzed by paired-end amplicon Next-Gen Sequencing (NGS) (Genewiz) with an average depth of 63,841 raw reads per sample (range 18,674 – 114,191 reads). Raw fastq files were processed by the CRISPResso2.0 pipeline with default settings to determine the location and frequencies of mutations as well as the insertions/deletions (indel) size^21^. To refine the analysis specifically to deletions proximal to the cut-site, the CRISPResso2 alignment output was further processed in Excel (See Table S1 for details). This approach discarded nucleotide substitutions, insertions, and erroneous indel-calling on specific SNPs present in the amplicons of Pbrm1 and Setd2 genomic regions.

**Table 2:** Primers for amplicon next generation Sequencing (NGS).

	Sense	Anti-sense	Size
Bap1	TGATAGGCTTCTGGGGTTTG	TCTAGCGCTTCTTCCTGCTC	384 bp
Pbrm1	GATTGCTGTGTGCCATGAAC	CAACTCCTGCAACAACTCCA	496 bp
Setd2	CTGTTTGAAGCTGGGCATAGA	CATTGCTAAGCGCAGTGAGA	423 bp
hU6-F	ACACTCTTTCCCTACACGACGCTCTTCCGATCTgactatcatatgcttaccgt		237 bp
gRNA-R	GACTGGAGTTCAGACGTGTGCTCTTCCGATCTCCGACTCGGTGCCACTT		

### Engineering of c3GIC9n-BPS murine embryonic stem cells

KH2 mouse ESCs were nucleofected with targeting plasmid DNA (c3GIC9n-BPS) and the pCAGGS-FLPe Flp-recombinase expression vector (Addgene #20,733)^22^. The transfected cells were seeded on DR4 feeder cells for overnight recovery, after which the media was replenished with hygromycin^23^. Resulting colonies were expanded and genotyped for targeting of the *Col1a1* locus using the Col1A1( +)/Col1A1(-) and Col1A1( +)/SAdpA(-) primer-pairs (wild type and transgenic, respectively), and three independent c3GIC9n-BPS ESC clones were obtained by targeting the inducible CRISPR plasmid into the *Col1a1* locus (Tables 3 and 4 for details). Copy-number PCR confirmed unique integration of the transgenic cassette. Robust induction of GFP and gene editing upon dox treatment was confirmed in one clonal line. Analysis of dox-treated mouse ESCs using the acrylamide DNA gel and the Surveyor/T7E1 assay confirmed that the gRNAs can induce mutations in the expected region.

**Table 3:** Genotyping primers.

Primer	Sequence
Col1A1( +)	AATCATCCCAGGTGCACAGCATTGCGG
Col1A1(−)	ACTTGAGGGCTCATGAACCTCCCAGG
SAdpA(−)	ATCAAGGAAACCCTGGACTACTGCG
Cas9_oIMR9020	AAGGGAGCTGCAGTGGAGTA
Cas9_24500	CAGGACAACGCCCACACA
Cas9_38651	TCCCCATCAAGCTGATCC
Cas9_38652	CTTCTTCTTTGGGGCCATCT
Cas9_Flox	CCTGGGCAACGTGCTGGTTATT
tta507_F	GCTGCTTAATGAGGTCGG
tta507_R	CTCTGCACCTTGGTGATC
CreER.1149.R	CAAGGCAGGGCTATTCTTCTTAGTG
CreER.910.F	GTTTCAATACCGGAGATCATGCAAG
hHIF1aP564A-F	TTGGAGATGTTAGCTGCCTATATCCC
hHIF1aN803A-R	AACTTCACAATCATAACTGGTCAGC

**Table 4:** PCR products for genotyping.

Forward	Reverse	gDNA	Description
Col1A1( +)	Col1A1(−)	220	Col1a1-WT
Col1A1( +)	SAdpA(−)	295	Col1a1-Integration
tta507_F	tta507_R	507	GGT-tTA
Cas9_oIMR9020	Cas9_24500	241	R26-WT
Cas9_38651	Cas9_38652	214	R26-Integration
CreER.910.F	CreER.1149.R	249	GGT-CreER
hHIF1aP564A-F	hHIF1aN803A-R	732	TRACK

### Generation of transgenic BPS-TA and BPS-Cre mice

The ESC clone was injected into C57BL/6 mouse embryos at the New York University mouse facility to create chimera mouse lines. All the founder mice were subsequently transferred and housed at the Research Animal Resource Center (RARC) of Weill Cornell Medicine and treated under a protocol approved by the local Institutional Animal Care and Use Committee (IACUC). All the mouse lines were maintained by the RARC, which provided staff and standardized housing facilities to ensure humane care of animals used for this research according to the Animal Welfare Act (updated guideline, National Research Council U.S.A, 2010). The founder mice with a c3GIC9n-BPS allele (BPS; Bap1-Pbrm1-Setd2) were identified, and cross-bred with two established transgenic mouse lines, the TRACK *ggt-HIF1A-M3* transgenic mice^13^, and a tet-Transactivator (tTA) driven by a 2.7 kb *γ-glutamyltransferase 1* (ggt) promoter, the ggt-tTA line^18^. The tTA (Tet-Off system) in this model is driven by the ggt promoter, which is active in the proximal tubules of the kidneys and restricts the expression of Cas9^D10A^ to the kidneys and testis. The in vivo expression of Cas9^D10A^ can be repressed by supplementation of the drinking water with doxycycline (2.0 mg/ml). The *ggt-HIF1A-M3* transgene encodes a constitutively active mutant HIF1A and was shown to induce features of early stage ccRCC^13^. The triple transgenic mice (ggt-tTA, TRACK, BPS) are referred to as BPS-TA mice. To replace the tTA-driven Cas9^D10A^ nickase with a Cre-driven Cas9^WT^ (relying on single rather than paired gRNAs), a breeding program was undertaken to introduce ggt-CreER and LSL-Cas9-P2A-GFP alleles, while removing the ggt-tTA allele from the BPS-TA line. The ggt-CreER facilitates LoxP recombination in response to tamoxifen treatment (intraperitoneal injection of 2 mg tam dissolved in 200 μl cottonseed oil)^24,25^. The LSL-Cas9-P2A-GFP allele provides expression of a Flag-tagged version of the humanized *S. Pyogenes* Cas9 (and GFP) in response to CreER activation by tam^26^. The quadruple transgenic mice (LSL-Cas9-P2A-GFP, ggt-CreER, TRACK, BPS) are referred to as BPS-Cre mice. Note that the BPS-Cre mice also harbor an GFP-IRES-Cas9^D10A^ allele, which remains silent in the absence of a tet regulator. The LSL-Cas9-P2A-GFP line was genotyped with primers Cas9_24500/Cas9_oIMR9020 (WT: 241 bp) and Cas9_38652/Cas9_38651 (TG: 214 bp). Allelic copy number of TRACK, ggt-tTA, and ggt-CreER alleles were determined by real-time qPCR on a MyiQ2 PCR machine using primer-pairs (hHIF1aP564A-F/hHIF1aN803A-R, tta507_F/tta507_R, and CreER.910.F/CreER.1149.R, respectively) (Tables 3 and 4). Phenotypic studies were performed in randomly selected male mice (BPS-TA, n = 19, BPS-Cre, n = 9) that met the predefined genotype criteria with homozygous presence of the c3GIC9n-BPS, HIF1A-M3 and ggt-tTA genes. The sample size of this study was based on previously published work which demonstrated a high incidence of malignant lesions in the kidneys using other gene editing technologies. All mice were monitored by the RARC staff and the investigators for the development of general health defects and adverse events during the experiments. All mice were euthanized without any prior drug treatments by either cervical dislocation or carbon dioxide euthanasia at random time points to perform phenotypic evaluation of the kidneys as well as other organs. Autopsy of animals was performed under sterile surgical conditions in our lab. Primary endpoint of the study was malignant transformation in the kidney, as assessed by a genito-urinary (GU) pathologist. Our study predominantly consists of a qualitative description of the aberrations that we detected in our mouse models as compared to genomic wild type kidneys. We used the Student’s T-Test and Analysis of variance (ANOVA) to determine statistical significance of quantitative differences.

For the high-fat experiment all mice were fed a regular chow diet after weaning for 1 to 2 weeks, after which they were randomly assigned to receive a regular chow (#5053, Lab Diet) or high fat diet (#58v8, Test Diet). The high fat diet contains 23.6% fat by weight (versus 5% in the regular diet), is rich in saturated fatty acids (9.05% versus 0.78%) and monounsaturated fatty acids (9.32% versus 0.96%), and it has increased amounts of polyunsaturated fatty acids. In one experiment BPS-TA mice (N = 3) were fed a high fat diet for 4 months prior to sacrifice.

### Transcriptome RNA sequencing

Kidney cortex was dissected from wild type mice (N = 7 kidneys) and BPS-TA mice (N = 4 kidneys) and stored in RNAlater. RNA was extracted using the RNeasy RNA purification kit (#74,134, Qiagen). RNA quality was assessed using a Bioanalyzer 2100 and only samples with RIN values > 9.5 were used for sequencing. The complete transcriptomes were sequenced using an Illumina HiSeq2000 Sequencer with 51 bp single-end reads. Differential expression analysis of genes, including statistical testing, was performed with the DESeq package (version 1.32, Bioconductor) in R. The Ingenuity Pathway Analysis software platform and Integrated Motif Activity Response analysis was used with default settings to detect altered pathways and transcription factor activity^27,28^. All genes with a q-value < 0.05 were considered differentially expressed from a statistically significant point of view.

### Histology and immunofluorescence

Immunostaining and Hematoxylin/Eosin (H&E) staining of the mouse kidney cortex sections was performed as previously described^29,30^. In brief, paraformaldehyde-fixed kidney sections were cut to 7 μm and mounted on glass slides by the Cornell Core Facility. Following deparafinization in Histoclear-II and unmasking by pressure cooker, boiling for 5 min in citrate buffer (#H-3300, Vector Labs), the sections were stained with primary antibody (Flag; GenScript #A00187S, lot-17G001273, GFP; St.John Labs #STJ140006, lot-000666270214) in a 1:200 dilution overnight at 4 °C. The staining was detected using Alexa Fluor Plus secondary antibodies in a 1:1,000 dilution 1 h at room temperature (anti-Goat, Invitrogen, #A32814, lot-UI289709, anti-Mouse, Invitrogen, #A32744, lot-TI271723). The sections were visualized using a Nikon TE2000 inverted microscope equipped with NIS Elements.

### Matrix-assisted laser desorption ionization imaging mass spectrometry (MALDI-IMS)

Kidney samples were snap-frozen in 2% carboxymethyl-cellulose, cryosectioned, and then mounted on indium tin oxide coated glass slides. MALDI-IMS was then performed as described (Laursen et al., 2021).

All experiments involving mice have been approved by the Weill Cornell IACUC Committee. All methods were carried out in accordance with relevant guidelines and regulations. All methods are reported in accordance with ARRIVE guidelines.

## Results

### Doxycycline inducible genome editing in *Bap1* and *Pbrm1*

**Figure 1:**
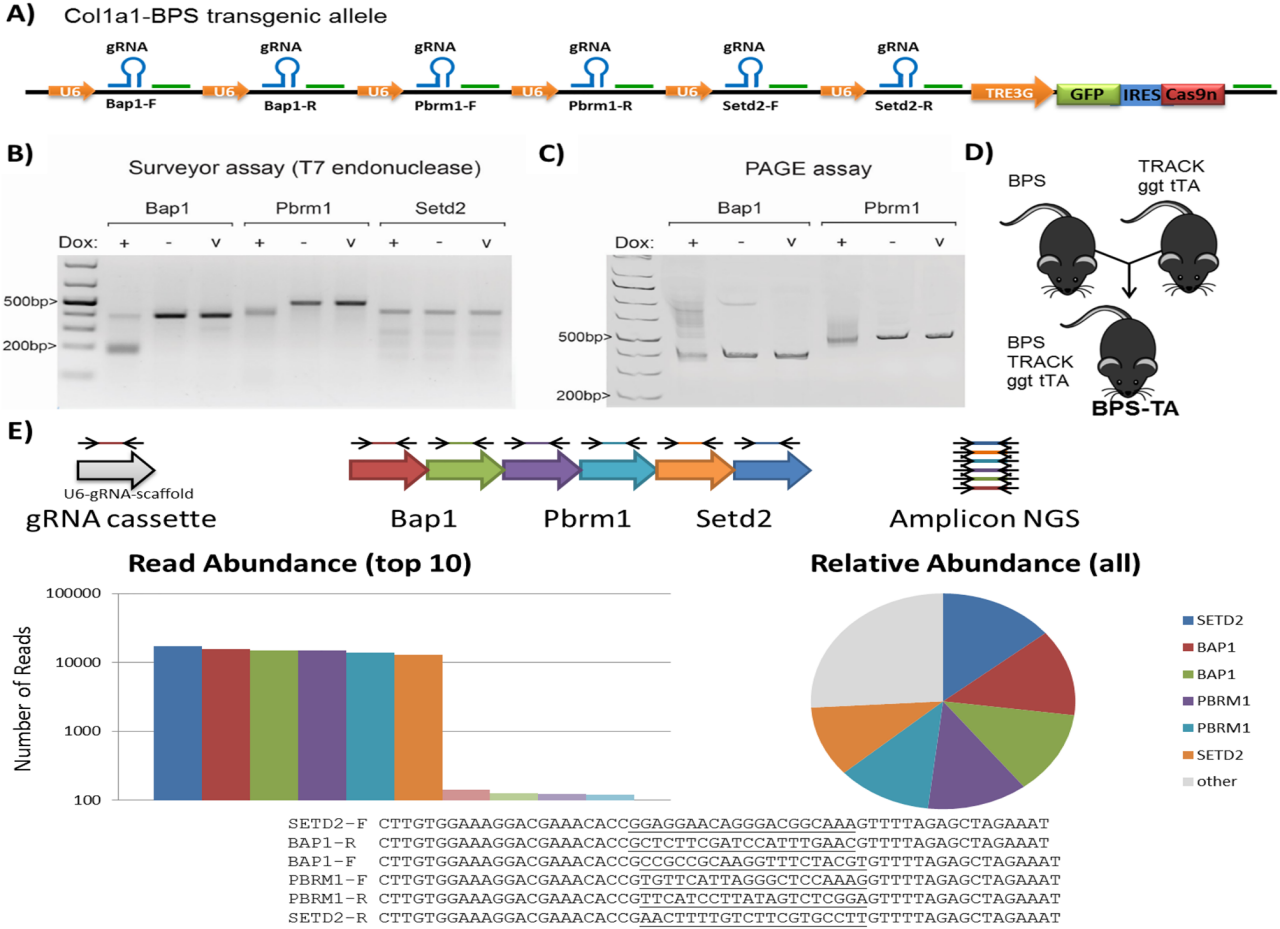
A doxycycline-inducible system for inducing somatic mutations in *Bap1*, *Pbrm1* and *Setd2*. (**A**) The *Col1a1* locus of mouse ESCs was targeted through recombination-mediated cassette exchange to harbor a dox-responsive GFP-Cas9^D10A^ expression vector and paired gRNAs for the targeting of mouse *Bap1*, *Pbrm1* and *Setd2*. (**B**) Surveyor assay for assessing the targeting of mouse *Bap1*, *Pbrm1*, and *Setd2* in the engineered ESCs. The ESCs express a reverse Tet-Transactivator (rtTA, Tet-On) from the modified R26 locus, R26/rtTA, which confers dox-inducible expression of the GFP-Cas9^D10A^ cassette (“v”; no T7E1 control,”- “; T7E1 treated PCR product derived from vehicle treated ESCs, ” + “; T7E1 treated PCR product derived from dox treated ESCs. (**C**) Gel retardation PAGE for assaying the targeting of mouse *Bap1* and *Pbrm1* in the engineered ESCs. (**D**) The validated ESCs were injected into blastocysts for tetraploid complementation, and one of the resulting founder mice was crossed with the TRACK/ggt-tTA double-transgenic mouse line to create triple-transgenic animals (BPS-TA). (**E**) The integrity of each individual gRNA expression cassette was confirmed by PCR amplification on genomic DNA from a BPS-TA mouse. The resulting PCR products were analyzed by Amplicon NGS. The six most prevalent reads perfectly matched the sequences of the six gRNAs, thus confirming that all six gRNA cassettes are maintained in the BPS-TA mouse line. The transgene overview and figure panels was generated and compiled in Microsoft Powerpoint software.

We designed six paired gRNAs that target the first exons of *Bap1*, *Pbrm1* and *Setd2* genes and cloned expression cassettes for these gRNAs into the c3GIC9n construct upstream of the TRE3G-GFP-IRES-Cas9^D10A^ cassette (Fig. 1A). We then transfected this construct together with a Flp-recombinase expression vector (pCAGGS-FlpE) into KH2 mouse ESCs, which allowed Flp-mediated insertion into the modified Collagen type 1 α1 (*Col1a1*) locus. Positive clones we tested for dox-regulated GFP expression, induced by the R26/rtTA allele present in the KH2 ESCs^22^. We extracted genomic DNA from GFP-positive clones, and PCR-amplified the *Bap1, Pbrm1* and *Setd2* target regions. These PCR products were first analyzed with the Surveyor/T7E1 assay, where treatment with the T7E1 endonuclease induced efficient digestion of *Bap1* and *Pbrm1* PCR products from dox-treated ESCs, as evident by the smaller bands **(**Fig. 1B). Since we did not observe digestion of *Bap1* and *Pbrm1* PCR products from vehicle treated ESCs, these results indicate that the amplified regions each contained hetrologous DNA, i.e. edited sequences. We did not detect any digestion of *Setd2* PCR products from dox-treated ESCs, suggesting that the *Setd2* target was not edited in these particular settings. To confirm the presence of genome edits, we analyzed the *Bap1* and *Pbrm1* PCR products in the polyacrylamide gel electrophoresis (PAGE) assay. The retarded gel migration, evident by an upward smearing, confirmed the presence of hetero-duplexes between WT and edited *Bap1* and *Pbrm1* specifically in dox-treated ESCs (Fig. 1C). These results confirmed that the BPS construct efficiently induces genome edits in *Bap1* and *Pbrm1*, but not in *Setd2*, in a dox-dependent manner.

Upon confirming the genome editing we injected these ESCs into mouse blastocysts for tetraploid complementation and obtained nine founder BPS (Bap1-Pbrm1-Setd2) mice. These founder mice were bred with TRACK/ggt-tTA double transgenic mice to generate the BPS-TA line (Fig. 1D). Importantly, since the tTA dissociates from the TRE3G promoter in the presence of dox, the BPS-TA line will induce expression of the Cas9^D10A^ transgene specifically in the *absence* of dox. Note that all BPS-TA mice evaluated in the subsequent analysis were biallelic for each of the three transgenes (ggt-tTA, TRACK, BPS). The integrities of the gRNA expression cassettes were confirmed by PCR amplification on genomic DNA from a BPS-TA mouse (Fig. 1E).

### Tissue specific genome edits of *Bap1* and *Pbrm1* in BPS-TA mice

**Figure 2:**
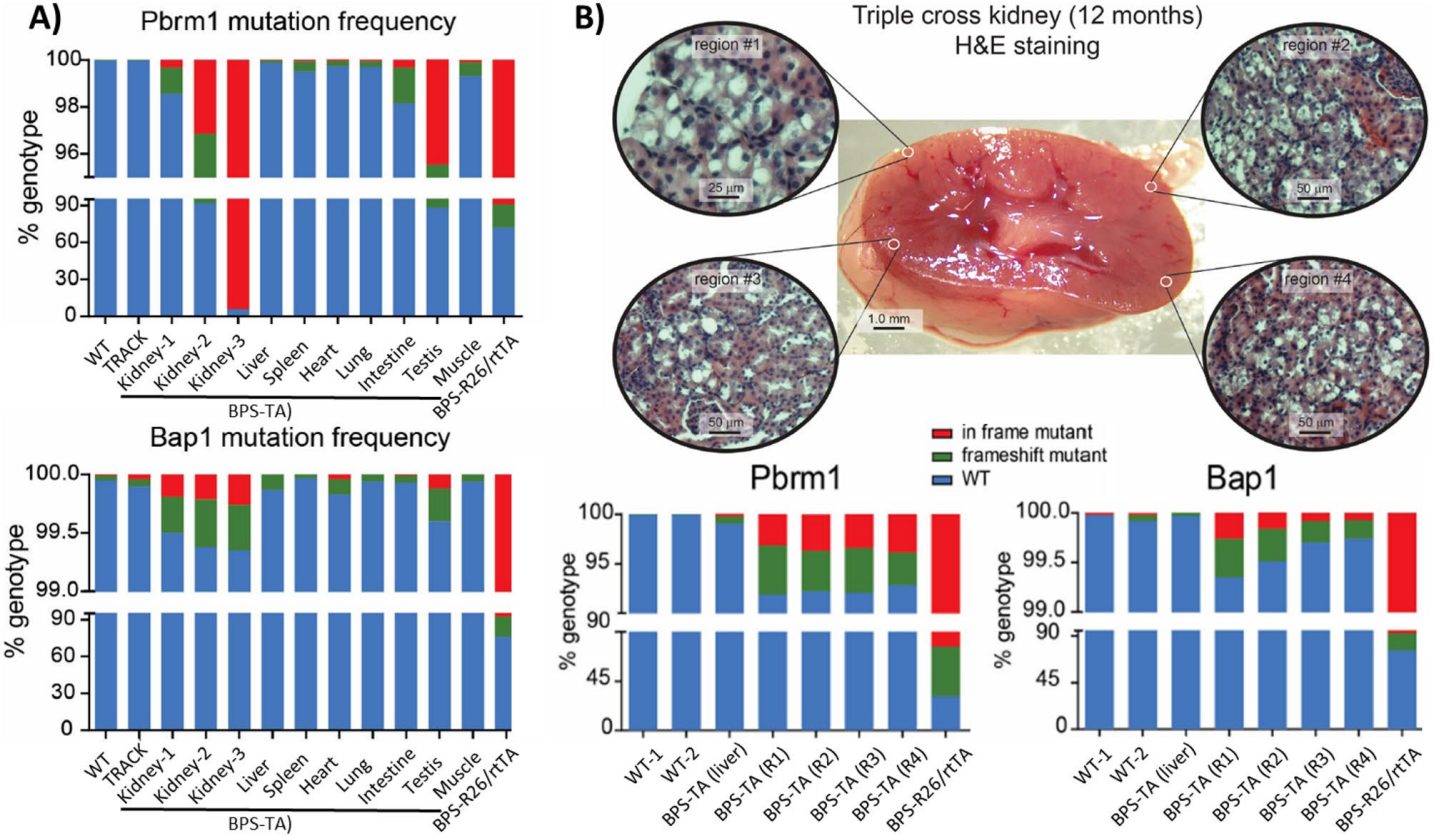
Organ- and sequence specific induction of mutations in Bap1 and Pbrm1 in BPS-TA mice. (**A**) Mutation frequencies in kidneys, liver, spleen, heart, lung, intestine, testis, and muscle of BPS-TA mice. Kidneys from WT and TRACK mice provided negative controls, while the intestines from dox-treated transgenic founder animals (BPS-R26/rtTA) served as positive control for genome editing. Pbrm1 and Bap1 are shown at the top and the bottom, respectively. (**B**) Mutation frequencies in different regions of BPS-TA kidneys. Livers from BPS-TA mice and WT kidneys provided negative controls, while the intestines from dox-treated transgenic founder animals (BPS-R26/rtTA) served as positive controls for genome editing. Overall morphology and histology of a BPS-TA kidney and the different regions selected for analysis are shown at the top. Pbrm1 and Bap1 are shown at the bottom left and the bottom right, respectively. Note that all BPS-TA mice evaluated were biallelic for each of the three transgenes. The images and figure were compiled in Adobe Illustrator software.

In order to evaluate genome editing of *Bap1* and *Pbrm1* in the BPS-TA mice, we isolated genomic DNA from the kidneys as well as liver, spleen, heart, lung, intestine, testis, and skeletal muscle and performed Amplicon NGS of the target regions (Fig. 2A**)**. We evaluated tissues from three BPS-TA animals (N = 3, aged up to 13 months), and included kidneys from WT and TRACK animals as negative controls (one each). For positive controls we utilized founder mice, which in addition to the BPS allele harbor a R26/rtTA allele^22^. When R26/rtTA mice are provided with dox supplemented drinking water, the R26/rtTA allele, which encodes expression of the reverse tet-transactivator (rtTA, Tet-On), causes a potent activation of the TRE3G promoter in the intestine, skin, and thymus^17^. The intestines from the BPS founder mice consequently served as positive controls for genome editing (Fig. 2A).

This genome editing analysis showed increased frequencies of somatic mutations in *Bap1* and *Pbrm1* in the kidneys of BPS-TA mice as compared to the kidneys from WT or TRACK mice. We detected low frequent in frame and frameshift mutations in *Bap1* and *Pbrm1* predominantly in the kidneys and testes from these mice, but not in the other organs. In a single BPS-TA animal (#3) we detected a highly frequent 12 nucleotide in-frame deletion in *Pbrm1*. Collectively, these results demonstrate that the introduction of the BPS and ggt-tTA transgenes in the TRACK model induces a low frequency of somatic mutations in *Bap1* and *Pbrm1* in the kidneys and testes of these mice. Despite the observed genome editing, we did not detect formation of visible renal tumors in these animals.

### Consistent levels of *Bap1* and *Pbrm1* genome edits in different regions of the BPS-TA kidney cortex

To investigate the impact of *Bap1* and *Pbrm1* indels on the malignant transformation of TRACK lesions in the kidneys, a cohort of BPS-TA mice was sacrificed (N = 10, aged 3 to 13 months). The kidneys, as well as various other organs, including livers, lungs and bones, were analyzed for macroscopic abnormalities or signs of malignancy. In this cohort we did not observe any significant changes in total body weight, kidney size, or tumor formation, while all the kidneys did show the hallmarks of the TRACK phenotype, with abundant clear cell formation, disorganized tubular structures and nuclear atypia (Fig. 2B**, top**). To determine the variability of the genome editing throughout the kidney, four spatially separated regions were sampled in a BPS-TA mouse (N = 1, 12 months old). No significant macroscopic or microscopic differences were observed in this mouse between the different regions (Fig. 2B**, top**). Similarly, a consistent percentage of 7.8% (range 7.2 – 8.1%) and 0.4% (range 0.3 – 0.7%) of the *Pbrm1* and *Bap1* amplicons was shown to be mutated in these regions, respectively (Fig. 2B**, bottom**). The intestines from the BPS founder mice again served as positive control for genome editing (Fig. 2B). These results indicate that the low frequencies of *Bap1* and *Pbrm1* mutations are not likely caused by a sampling error. In addition, these results suggest that the gene edits are insufficient to introduce malignant transformation of TRACK lesions within the evaluated time frame.

### Metabolic stress conditions do not introduce clonal expansion of gene-edited cells

**Figure 3:**
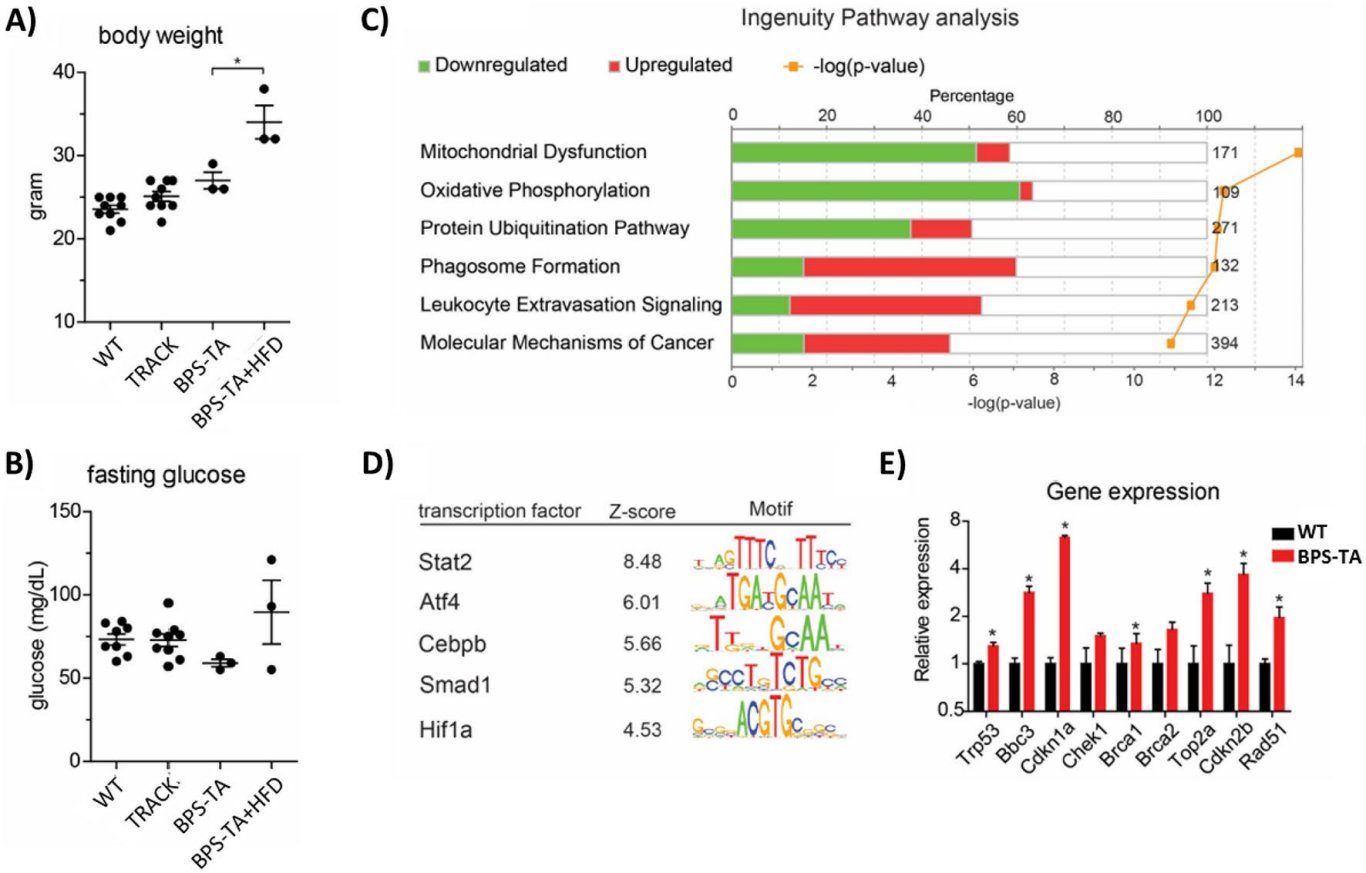
Activation of the immune and DNA damage response pathways in the kidney of BPS-TA mice. (**A**) BPS-TA mice on a high fat diet showed increased weight relative to chow fed BPS-TA. No significant differences in body weight were noted between BPS-TA, WT, and TRACK mice on a regular chow diet. (**B**) BPS-TA mice on a high fat diet showed a non-significant trend of increased fasting glucose levels relative to chow fed BPS-TA, confirming induction of metabolic stress. No significant differences in fasting glucose levels were noted between BPS-TA, WT, and TRACK mice on a regular chow diet. (**C**) Pathway Analysis of differentially expressed genes ‘Mitochondrial dysfunction’, ‘Oxidative Phosphorylation’, ‘Protein Ubiquitination Pathway’, ‘Phagosome Formation’, ‘Leukocyte extravasation Signaling’ and ‘Molecular Mechanisms of Cancer’ were the most significantly different pathways between WT and BPS-TA kidneys. The analysis was performed using Ingenuity Pathway Analysis software on RNAse dataset. Genes with (q < 0.05) were considered differentially expressed between BPS-TA (N = 4) and WT kidneys (N = 7) on a chow diet. (**D**) Promoter motif analysis indicated that Stat2, Atf4, Cebpb, Smad1 and Hif1a are significantly activated in BPS-TA kidneys as compared to WT kidneys. E) Significantly increased transcript levels of Trp53, Bbc3, Cdkn1a, Brca1, Top2a, Cdkn2a and Rad51, as measured by RNAseq. These results indicate that activation of the immune and DNA damage response pathways are potential mechanisms by which BPS-TA mice suppress carcinogenesis in the kidneys. Note that all BPS-TA mice evaluated were biallelic for each of the three transgenes.

Previous research has revealed that *Bap1* and *Pbrm1* loss of function mutations do not necessarily enhance proliferation, but particularly provide a growth advantage under stress conditions^31,32^. To investigate whether metabolic stress conditions could promote the clonal expansion of TRACK lesions (thereby indirectly enhancing the *Bap1* and *Pbrm1* mutation frequencies), BPS-TA mice (N = 6) were randomized to receive either a regular chow or high fat diet. The mice that were fed with a high fat diet for four months became obese (Fig. 3A) and showed a non-significantly increased fasting glucose level (Fig. 3B). We did not detect tumors in the kidneys of these BPS-TA mice. We evaluated the *Bap1* and *Pbrm1* target regions in both kidneys from each mouse, and similar to previous cohorts, we detected low frequencies of mutations in both genes (Figure S1). We did not detect any statistically significant differences in the *Bap1* and *Pbrm1* indel frequencies between mice on a regular chow or high fat diet. These results indicate that metabolic stress conditions are not likely promoting the clonal expansion of TRACK lesions with *Bap1* and/or *Pbrm1* loss of function.

### Evidence of mitochondrial damage, leukocyte invasion, and activation of *Trp53* in BPS-TA kidneys

To gain insight in the causes of the low indel frequency in BPS-TA mice, we did transcriptome profiling of kidney cortices from WT (N = 7) and BPS-TA kidneys (N = 4) using RNAseq. In total 7775 transcripts showed statistically significant (q < 0.05) different expression levels between these two conditions, with 4014 upregulated and 3761 downregulated genes. Similar to our previous analyses, we detected increased mRNA expression levels of several well-known ccRCC genes, such as *Car9* (5.4 fold), *Hk2* (26.7 fold), *Ndufa4l2* (23.5 fold) and *Vegfa* (1.2 fold). To determine whether regulation of gene clusters with similar functional annotations occurred in the kidneys of BPS-TA mice, we performed ingenuity pathway analysis (Fig. 3C). The ‘Mitochondrial dysfunction’, ‘Oxidative Phosphorylation’ and ‘Protein Ubiquitination Pathway’ were highly significant pathways in BPS-TA kidneys that contained predominantly downregulated transcripts. The ‘Phagosome formation’, ‘Leukocyte Extravasation Signaling’ and ‘Molecular Mechanisms of Cancer’ pathways also showed significant regulation in BPS-TA kidneys, but contained predominantly upregulated transcripts. To investigate whether these changes were coordinated by specific transcription factors, we performed a genome wide promoter motif analysis (Fig. 3D). This analysis indicated that the immune-associated transcription factors *Stat2, Cebpb* and *Smad1* were activated in the kidneys of BPS-TA mice. Similar to our previous analyses, we also detected significant activation of *Atf4* and *Hif1a* signaling^14^. These results suggest activation of mitochondrial stress and immune responses in BPS-TA kidneys.

Recent research showed that the induction of double-strand breaks by Cas9 can be toxic and may activate p53 in certain cells and tissues^33,34^. We investigated whether activation of p53 pathways occurred in BPS-TA kidneys (Fig. 3E). Indeed, we detected increased mRNA expression levels of *Trp53* and its target genes, *Bbc3*/*Puma* and *Cdkn1a*/*p21*^*waf*^. In addition, several other genes that have previously been associated with the DNA damage response showed elevated expression levels in the BPS-TA kidneys, including *Brca1*, *Top2a*, *Cdkn2b*, and *Rad51*. Collectively, these results suggest activation of the immune system and the DNA damage response as potential tumor suppressive mechanisms in the kidneys of BPS-TA mice.

### BPS-TA kidneys exhibit decreased levels of amino acids and purines and increased levels of cholesterol-sulfate

**Figure 4:**
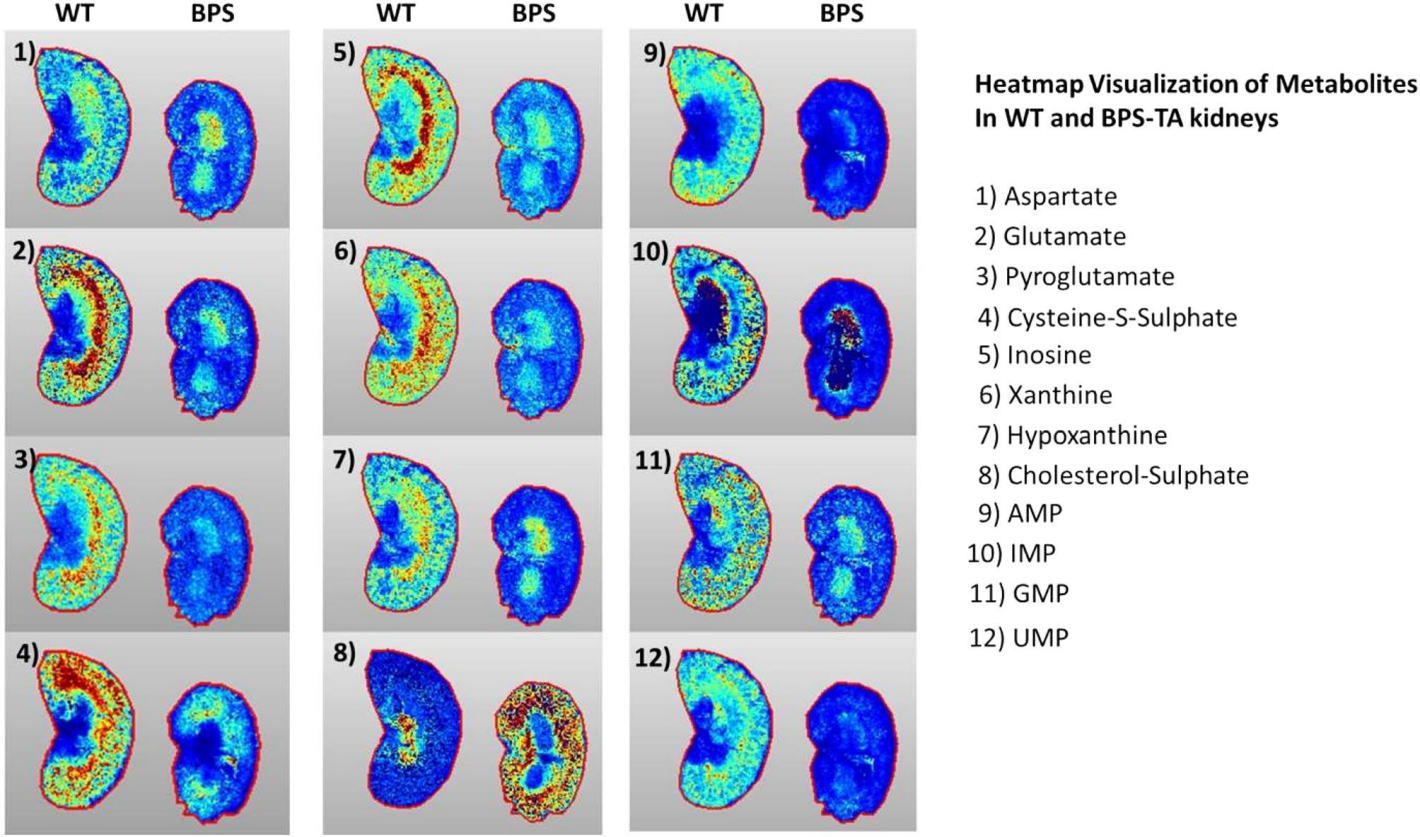
Spatial Distribution of Amino Acids, Purines, and Cholesterol Sulfate in the Wt vs. BPS Kidneys by MALDI-IMS. Heatmaps indicate the relative levels of various purines, amino acids, and cholesterol sulfate imaged from two mice of each genotype. Quantification as determined by MALDI-IMS. Note that red = a high level compared to deep blue, a low level of each metabolite. This figure was compiled in Microsoft Powerpoint.

In order to further characterize the metabolic alterations in the BPS kidneys, we next used MALDI-IMS to assess the levels of various metabolites in WT vs. BPS-TA kidneys (3 months of age). We detected decreased levels of aminoacids aspartate and glutamate, decreased levels of the purines hypoxanthine, inosine, and xanthine, while cholesterol sulfate levels were increased in the BPS-TA compared to WT kidneys (Fig. 4). These data support the transcriptome profiling in demonstrating that the BPS-TA kidneys show very different metabolic features compared to WT kidneys. Importantly, cholesterol sulfate was demonstrated to enhance T-cell evasion (PMID: 35094065), hinting of a tumor-facilitating microenviroment of the BPS kidneys.

### Modulating the gene editing strategy for increased targeting efficiency

**Figure 5:**
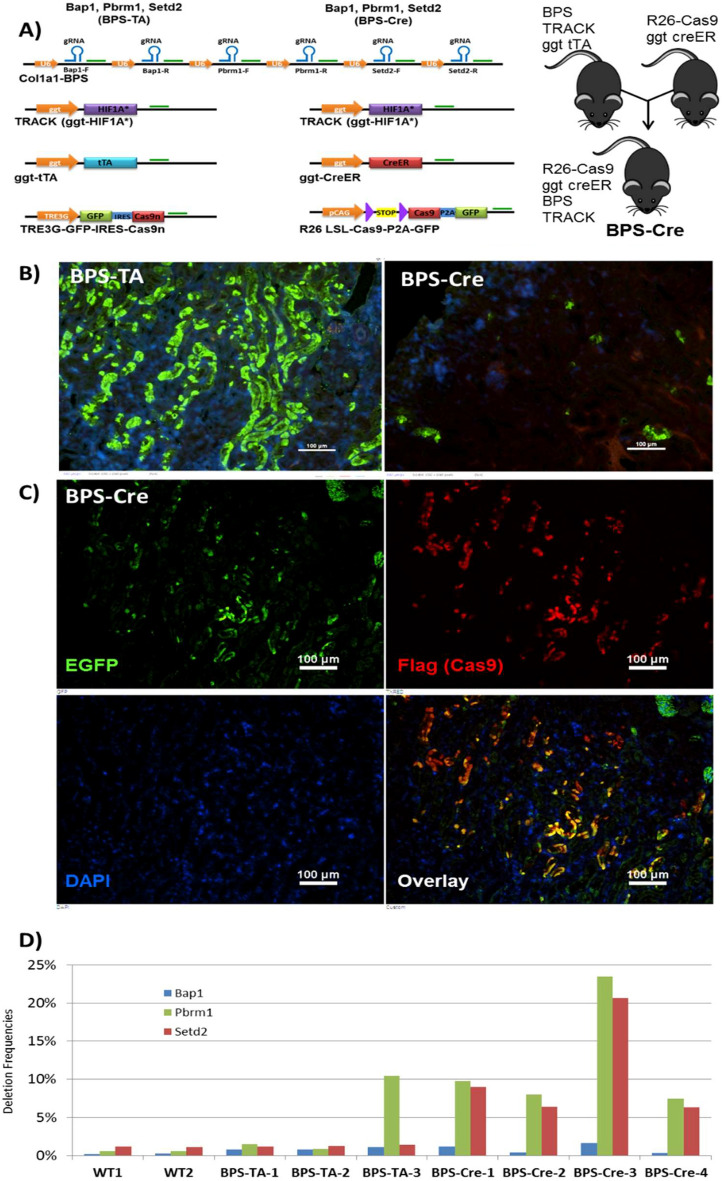
Cre-regulated perturbation of Bap1, Pbrm1, and Setd2 in BPS-Cre mice. (**A**) For the initial targeting of mouse Bap1, Pbrm1, and Setd2 we employed a dox-responsive GFP-IRES-Cas9^D10A^ allele, whereas in a revised approach we employed a cre-regulated Cas9-P2A-GFP (left and right, respectively). Ggt promoters drive tissue specific expression of the Cas9 activator (tTA and Cre, respectively), and facilitated gene perturbation specifically in the proximal tubules of the kidneys of the TRACK ccRCC mouse model (BPS-TA and BPS-Cre animals). (**B**) Immunostaining of GFP served as a marker for proximal tubule cells with active Cas9. Note the more pervasive expression using tTA regulation compared with Cre regulation (left and right, respectively). Magnification; × 200, scale bar; 100 μm. Representative images are shown. (**C**) Co-immunostaining of GFP and the Flag epitope of Cas9 (green and red, respectively) demonstrates co-expression of the two proteins, and validates GFP as a proxy for Cas9 expression. Magnification; × 200, scale bar; 100 μm. Representative images are shown. D) Frequencies of on-site deletions in Bap1, Setd2, and Pbrm1 in BPS-TA and BPS-Cre kidneys. WT kidneys served as negative controls. Note that average deletion frequencies were increased in BPS-Cre vs BPS-TA kidneys for Pbrm1 (12.2% vs. 4.3%) and Setd2 (10.6% vs. 1.3%), whereas deletion frequencies for Bap1 was unchanged in BPS-Cre vs BPS-TA kidneys (0.9% vs. 0.9%), but higher than background levels (WT kidneys; 0.3%). Note also that one animal (#3) among the BPS-Cre animals had the highest levels of gene editing of Bap1, Pbrm1, and Setd2 (1.6%, 23.4%, and 20.7%, respectively). Note that all BPS-TA mice evaluated were biallelic for each of the three transgenes, and all BPS-Cre mice were biallelic for each of the four transgenes. The transgene overview and figure panels was generated and compiled in Microsoft Powerpoint software.

Because the frequencies of indels in *Pbrm1* and *Bap1* were low in our first BPS-TA model, we then generated a second model in which Cas9^WT^ (hSpCsn1), rather than Cas9^D10A^ (hSpCsn1n), is introduced into the renal cortex. The purpose of this change was to increase the frequencies of indels and to obtain indels in the *Setd2* gene. The BPS-Cre mice were generated as described in detail in the Materials and Methods section (Fig. 5A). Note that all BPS-Cre mice evaluated in the subsequent analysis were biallelic for each of the four transgenes (LSL-Cas9-P2A-GFP, ggt-CreER, TRACK, BPS). Unexpectedly, the BPS-TA mice showed much stronger and more widespread staining for GFP than the BPS-Cre mice (Fig. 5B). Co-staining for GFP and the Flag-epitope of Cas9^WT^ confirmed the use of GFP as a proxy for Cas9 expression (Fig. 5C). We next evaluated deletion frequencies in BPS-TA and BPS-Cre kidneys and observed much higher genome editing of *Pbrm1* and *Setd2* in the BPS-Cre (N = 4) compared to the BPS-TA mice (N = 3) (Fig. 5D). Two WT kidneys were included as negative controls. These data suggest that in terms of generating indels in the three tumor suppressor genes in these ccRCC models, the Cre-regulated Cas9^WT^ is more potent than the tTA-regulated Cas9^D10A^ (See Figures S2 and S3 for further details). Additionally, the chicken actin promoter (CAG), which drives the Cas9^WT^ expression, may be a stronger promoter in the proximal tubule cells than the TRE3G that drives the Cas9^D10A^ expression (Fig. 5A). Despite the enhanced genome editing, we did not detect macroscopic renal tumors in the BPS-Cre animals (N = 5 evaluated at 3–9 months of age).

## Discussion

Inducible CRISPR-Cas9 systems have revolutionized the development of GEMMs and allow flexible and fast introduction of loss-of-function mutations in various genes in a tissue-specific and dox-dependent manner. Here we used this technology to generate a model for ccRCC, which in humans is caused in part by the biallelic loss of various tumor suppressor genes, including *VHL*, *BAP1, PBRM1,* and *SETD2*. These gene sequences are strongly conserved between mice and humans. Our results indicate that our novel BPS-TA GEMM indeed promotes the induction of early frameshift mutations in *Bap1* and *Pbrm1* in the kidney cortices of TRACK mice. However, these mutations did not enhance macroscopic tumor formation in the small cohorts of mice that we investigated here, possibly because of the low mutation frequencies. These low mutation frequencies may be related to the highly specific Cas9^D10A^, which requires binding of paired gRNAs to *both* complementary DNA strands before Cas9 induces double strand breaks^19^, used for the BPS-TA GEMM. We consider it likely that biallelic mutations occur in very small numbers of cells in BPS-TA kidneys and that these could still promote carcinogenesis, but the latency time of these tumors might be very long (> 13 months), which makes the model somewhat unpractical for drug development purposes. Previous research has suggested that mutations in *Bap1* and *Pbrm1* tend to occur in a mutually exclusive pattern and give rise to different types of tumors^35,36^. Future experiments with our BPS-TA model in which we use longer incubation times may shed more light on this and show potential divergent, clonal development of lesions with various combinations of in frame and frameshift mutations in *Bap1* and *Pbrm1*.

The presence of a competent immune system is an important asset of GEMMs that distinguishes these models from various other model systems, such as cell culture, organoids and orthotopic xenograft models. To gain insight into the mechanisms by which the BPS-TA mice might suppress the induction of mutations, we performed transcriptome profiling on a small series of kidney cortices. ‘Leukocyte Extravasation Signaling’ and ‘Phagosome Formation’ were among the significantly enriched pathways in the BPS-TA kidneys. An independent analysis also indicated that the transcription factors *Stat2*, *Cebpb* and *Smad1* are activated in BPS-TA kidneys. These transcription factors have been associated with interferon, TGF-β signaling, and maturation of dendritic cells and macrophages^37–39^. In line with previous research about CRISPR-Cas9 based genome editing, we also found evidence of p53 activation in BPS-TA kidneys, with elevated expression of target genes such as *Bbc3*/*Puma*, which induces apoptosis, and the cell cycle inhibitor *Cdkn1a*/*p21*^*wa*^^40,41^. These results are in line with previous investigations, which identified the adaptive as well as the innate immune system as important frontiers to cancer development in immunocompetent preclinical models^42–44^. More research, potentially involving short term treatment with immunosuppressive agents, could provide more insights into this potential mechanism of tumor suppression in BPS-TA mice.

We then decided that our first BPS-TA model system could benefit from some improvements. First, our initial design included *Setd2* as a target for genome editing, but our results with the BPS-TA mice showed no evidence of *Setd2* gene disruption in vivo (Figure S2. It is possible that *Setd2* loss of function mutations are detrimental to non-transformed cells and that mutated cells are rapidly lost, as observed for *Bap1*^31^, or the lack of *Setd2* editing could simply be attributed to a technical challenge. Second, for the first BPS-TA model we used a construct with a tetracycline-responsive promoter (TRE3G) to be able to “switch off” Cas9 expression in case there were toxic effects on kidney function. However, we did not observe any signs of kidney failure in our model, and hence we decided that the BPS construct could be simplified by replacing the TRE3G promoter by the constitutively active, truncated ggt promoter, which we showed has high activity in the proximal tubules of the kidney but limited activity in other organs. Third, we demonstrated that replacing the Cas9^D10A^ with Cas9^WT^ improved the gene editing efficiency (Fig. 5D). A fourth improvement in the BPS-Cre mice is the use of the constitutive actin promoter to drive Cas9^WT^ expression specifically in proximal tubule cells. Fifth, whereas the genome editing of the BPS-TA can be halted by supplementing the mice with dox, the Cas9 of the BPS-Cre animals is continuously expressed upon Cre-mediated excision of the stop cassette flanked by the loxP sites, and sixth, the Cas9^D10A^ is located downstream of GFP whereas the Cas9^WT^ is located upstream of GFP, which in the BPS-Cre model favors Cas9 expression over GFP.

Comparing the BPS-TA with the BPS-Cre transgenic mice, we found more extensive expression of GFP in the BPS-TA kidneys compared with those of the BPS-Cre mice, yet the genome editing was more efficient in the BPS-Cre mice (Fig. 5). The more extensive GFP expression in the BPS-TA animals may be attributed to GFP being upstream of Cas9 (see point six in the above paragraph). The Cas9^WT^ of the BPS-Cre animals requires only a single gRNA per target, whereas the Cas9^D10A^ of the BPS-TA animals requires paired gRNA for successful gene disruption. This may explain not only the higher edit frequencies of the BPS-Cre animals, but also the successful targeting of *Setd2* in the BPS-Cre kidneys. Indeed, when evaluating the specific locations of the *Setd2* genome edits in the BPS-Cre kidneys (Figure S4), we found edits proximal to Setd2-F site (but not near the Setd2-R site), suggesting that among the Setd2 gRNAs only Setd2-F is active. We suspect that the U6-driven polIII transcription of Setd2-R may be prematurely terminated due to the presence of a T_4_ in the targeting sequence (AACTTTTGTCTTCGTGCCTTTGG). In the BPS-Cre kidneys, the editing frequencies of *Bap1* remained low, hinting at adverse effects of Bap1 loss-of-function on survival of proximal tubule cells. We speculate that loss of *Bap1* could be tumorigenic specifically to check-point impaired cells. We noted overall morphology and histology of the BPS-Cre kidneys comparable to those of TRACK mice (Figure S5), suggesting that the gene edits do not support malignant transformation in our early-stage ccRCC mouse model (TRACK).

Lastly, we propose that the current model could benefit from inclusion of a biomarker for tumor development. For example, the secreted *Gaussia* luciferase would allow for non-invasive monitoring of tumor development in blood, as well as in vivo by use of bioluminescence imaging ^45^. This luciferase or other any marker could replace GFP in our construct, which is more useful to monitor promoter activity in cell culture.

One limitation of this study is that loss of actual gene function (*Bap1, Pbrm1,* and/or *Setd2*) cannot be conclusively demonstrated in these two models, BPS-TA and BPS-Cre. Future evaluations would require single cell RNA sequencing, with which we could evaluate the stochastic combinations of somatic inactivation of *Bap1, Pbrm1,* and *Setd2* in individual animals; this approach could provide insights into the step-wise ccRCC tumorigenesis. With respect to co-deletions, the cells that activate Cas9 are likely to edit multiple target sites simultaneously. This is supported by supplementary Figure S4, which demonstrates simultaneous edits of Pbrm1 -R and -F sites in multiple alleles (evident by both excision between both cut-sites and by the simultaneous presence of minor edits at both the -R and -F sites). Further research is needed to enable use of these two models for the development of novel therapeutics for ccRCC. We could introduce additional gRNAs that lead Cas9 to other targets, such as *Trp53* and/or *Cdkn2a*, which has been associated with the development of metastatic ccRCC in humans and other GEMMs^15,46^.

In conclusion, we successfully achieved somatic genome editing of *Bap1*, *Pbrm1*, and to some extent *Setd2* in two novel GEMMs for early ccRCC through use of an inducible CRISPR-Cas9 system. However, the relatively limited amount of *Pbrm1, Setd2,* and particularly *Bap1* genome editing in proximal tubules that we obtained with both of these models, BPS-TA and BPS-Cre, did not induce the formation of rapidly progressive and/or metastatic renal cancers in a small series of TRACK mice. Further studies are expected to increase the cellular resolution, e.g. by employing single-cell RNAseq to ascertain the effects of specific combinatorial gene inactivations. Additional genomic alterations, such as more extensive p3 deletions, inactivation of *Trp53* and/or *Cdkn2a* or overexpression of c-Myc, may synergize with the genomic perturbations in the BPS-TA and BPS-Cre models to induce tumor formation, and hypotheses such as these may be addressed in future studies.

### Link and accession no. for datasets taken from the The Cancer Genome Atlas (TCGA) consortium

We analyzed public transcriptomics and corresponding clinical datasets from the The Cancer Genome Atlas (TCGA) consortium. The results from these analyses were previously published in Nature, 499, 43–49 (2013). The KIRC datasets (version 2016_01_28) are available in a public repository that can be accessed through the Broad institute, Firehose website: Broad GDAC Firehose (broadinstitute.org). The murine datasets presented in this study can be found in online repositories. The names of the repository/repositories and accession number(s) can be found below: NCBI GEO database, accession number GSE199795.

## Supplementary Information

Supplementary Information.

## Data Availability

All datasets that were generated from mouse studies are included as supplementary files (datasets 1–5). In addition, we analyzed public transcriptomics and corresponding clinical datasets from the The Cancer Genome Atlas (TCGA) consortium. The results from these analyses were previously published in Nature, 499, 43–49 (2013)^2^. The KIRC datasets (version 2016_01_28) are available in a public repository that can be accessed through the Broad institute, Firehose website: Broad GDAC Firehose (broadinstitute.org). The murine datasets presented in this study can be found in online repositories. The names of the repository/repositories and accession number(s) can be found below: NCBI GEO database, accession number GSE199795.

## Abbreviations

GEMMs: Genetically engineered mouse models
HIF1A: TRAnsgenic Cancer of the Kidney, TRACK
ccRCC: Clear cell renal cell carcinoma
Cas9^D10A^: Nickase, hSpCsn1n
tet: Tetracycline
ggt: γ-Glutamyltransferase 1
indels: Insertions and deletions
dox: Doxycycline
tam: Tamoxifen
TRE3G: Tetracycline-responsive elements
ESCs: Embryonic stem cells
gRNAs: Guide RNAs
tTA, Tet-Off: Tet-transactivator
rtTA, Tet-On: Reverse tet-transactivator
GFP: Enhanced green florescent protein
hSpCsn1: Cas9^WT^
BPS: C3GIC9n-BPS allele
H&E: Hematoxylin and eosin
PAGE: Polyacrylamide gel electrophoresis
WT: Wild type
LSL: Lox-stop-lox
NGS: Next-gen sequencing
CAG: Chicken actin promoter

## References

[1] Mitchell TJ (2018). Timing the landmark events in the evolution of clear cell renal cell cancer: TRACERx renal. Cell.

[2] Network CGAR (2013). Comprehensive molecular characterization of clear cell renal cell carcinoma. Nature.

[3] Ricketts CJ (2018). The cancer genome atlas comprehensive molecular characterization of renal cell carcinoma. Cell Rep..

[4] Turajlic S (2018). Deterministic evolutionary trajectories influence primary tumor growth: TRACERx renal. Cell.

[5] Choueiri TK (2021). Adjuvant pembrolizumab after nephrectomy in renal-cell carcinoma. N. Engl. J. Med..

[6] Rini BI, Powles T (2019). Immune checkpoint blockade plus axitinib for renal-cell carcinoma Reply. N. Engl. J. Med..

[7] Motzer RJ (2018). Nivolumab plus Ipilimumab versus sunitinib in advanced renal-cell carcinoma. N. Engl. J. Med..

[8] Rankin EB, Tomaszewski JE, Haase VH (2006). Renal cyst development in mice with conditional inactivation of the von Hippel-Lindau tumor suppressor. Cancer Res..

[9] Wang SS (2014). Bap1 is essential for kidney function and cooperates with Vhl in renal tumorigenesis. Proc. Natl. Acad. Sci. U. S. A..

[10] Nargund AM (2017). The SWI/SNF protein PBRM1 restrains VHL-loss-driven clear cell renal cell carcinoma. Cell. Rep..

[11] Hoefflin R (2020). HIF-1α and HIF-2α differently regulate tumour development and inflammation of clear cell renal cell carcinoma in mice. Nat. Commun..

[12] Shenoy N (2020). HIF1α is not a target of 14q deletion in clear cell renal cancer. Sci. Rep..

[13] Fu L, Wang G, Shevchuk MM, Nanus DM, Gudas LJ (2011). Generation of a mouse model of Von Hippel-Lindau kidney disease leading to renal cancers by expression of a constitutively active mutant of HIF1alpha. Cancer Res..

[14] Fu L, Minton DR, Zhang T, Nanus DM, Gudas LJ (2015). Genome-wide profiling of track kidneys shows similarity to the human ccRCC transcriptome. Mol. Cancer. Res..

[15] Bailey ST (2017). MYC activation cooperates with Vhl and Ink4a/Arf loss to induce clear cell renal cell carcinoma. Nat. Commun..

[16] Harlander S (2017). Combined mutation in Vhl, Trp53 and Rb1 causes clear cell renal cell carcinoma in mice. Nat. Med..

[17] Dow LE (2015). Inducible in vivo genome editing with CRISPR-Cas9. Nat. Biotechnol..

[18] Fiaschi-Taesch NM (2004). Prevention of acute ischemic renal failure by targeted delivery of growth factors to the proximal tubule in transgenic mice: The efficacy of parathyroid hormone-related protein and hepatocyte growth factor. J. Am. Soc. Nephrol..

[19] Ran FA (2013). Double nicking by RNA-guided CRISPR Cas9 for enhanced genome editing specificity. Cell.

[20] Zhu X (2014). An efficient genotyping method for genome-modified animals and human cells generated with CRISPR/Cas9 system. Sci. Rep..

[21] Pinello L (2016). Analyzing CRISPR genome-editing experiments with CRISPResso. Nat. Biotechnol..

[22] Beard C, Hochedlinger K, Plath K, Wutz A, Jaenisch R (2006). Efficient method to generate single-copy transgenic mice by site-specific integration in embryonic stem cells. Genesis.

[23] Premsrirut PK (2011). A rapid and scalable system for studying gene function in mice using conditional RNA interference. Cell.

[24] Dworniczak B (2007). Inducible Cre/loxP recombination in the mouse proximal tubule. Nephron Exp. Nephrol..

[25] Iwano M (2002). Evidence that fibroblasts derive from epithelium during tissue fibrosis. J. Clin. Invest..

[26] Platt RJ (2014). CRISPR-Cas9 knockin mice for genome editing and cancer modeling. Cell.

[27] Krämer A, Green J, Pollard J, Tugendreich S (2014). Causal analysis approaches in ingenuity pathway analysis. Bioinformatics.

[28] Balwierz PJ (2014). ISMARA: Automated modeling of genomic signals as a democracy of regulatory motifs. Gen. Res..

[29] Tang XH, Knudsen B, Bemis D, Tickoo S, Gudas LJ (2004). Oral cavity and esophageal carcinogenesis modeled in carcinogen-treated mice. Clin. Cancer Res..

[30] Laursen KB (2021). Mitochondrial Ndufa4l2 enhances deposition of lipids and expression of Ca9 in the track model of early clear cell renal cell carcinoma. Front. Oncol..

[31] Chen P (2019). Loss of BAP1 results in growth inhibition and enhances mesenchymal-epithelial transition in kidney tumor cells. Mol. Cell Proteom..

[32] Gao W, Li W, Xiao T, Liu XS, Kaelin WG (2017). Inactivation of the PBRM1 tumor suppressor gene amplifies the HIF-response in VHL-/- clear cell renal carcinoma. Proc. Natl. Acad. Sci. U. S. A..

[33] Haapaniemi E, Botla S, Persson J, Schmierer B, Taipale J (2018). CRISPR-Cas9 genome editing induces a p53-mediated DNA damage response. Nat Med.

[34] Ihry RJ (2018). p53 inhibits CRISPR-Cas9 engineering in human pluripotent stem cells. Nat. Med..

[35] Peña-Llopis S (2012). BAP1 loss defines a new class of renal cell carcinoma. Nat. Genet..

[36] Hsieh JJ (2017). Genomic biomarkers of a randomized trial comparing first-line everolimus and sunitinib in patients with metastatic renal cell carcinoma. Eur. Urol..

[37] Xu J (2016). STAT2 is required for TLR-induced murine dendritic cell activation and cross-presentation. J. Immunol..

[38] Jaitin DA (2016). Dissecting immune circuits by linking CRISPR-pooled screens with single-cell RNA-seq. Cell.

[39] Nurgazieva D (2015). TGF-β1, but not bone morphogenetic proteins, activates Smad1/5 pathway in primary human macrophages and induces expression of proatherogenic genes. J. Immunol..

[40] Yu J, Wang Z, Kinzler KW, Vogelstein B, Zhang L (2003). PUMA mediates the apoptotic response to p53 in colorectal cancer cells. Proc. Natl. Acad. Sci. U. S. A..

[41] Bunz F (1998). Requirement for p53 and p21 to sustain G2 arrest after DNA damage. Science.

[42] Vesely MD, Kershaw MH, Schreiber RD, Smyth MJ (2011). Natural innate and adaptive immunity to cancer. Annu. Rev. Immunol..

[43] Shankaran V (2001). IFNgamma and lymphocytes prevent primary tumour development and shape tumour immunogenicity. Nature.

[44] Kaplan DH (1998). Demonstration of an interferon gamma-dependent tumor surveillance system in immunocompetent mice. Proc. Natl. Acad. Sci. U. S. A..

[45] Wurdinger T (2008). A secreted luciferase for ex vivo monitoring of in vivo processes. Nat. Methods.

[46] Turajlic S (2018). Tracking cancer evolution reveals constrained routes to metastases: TRACERx renal. Cell.

