# Supplementary material for: Novel genetically engineered mouse models for clear cell renal cell carcinoma
Source: Sci Rep. 2023 May 22;13:8246. doi: 10.1038/s41598-023-35106-7 (PMC10203127; doi:10.1038/s41598-023-35106-7)
Supplement: Supplementary file 1 — Supplementary Information. [file 41598_2023_35106_MOESM1_ESM.pdf]

Column	Legend	Formulas
A	Aligned_Sequence	CRISPResso2
B	Reference_Sequence	CRISPResso2
C	Reference_Name	CRISPResso2
D	Read_Status	CRISPResso2
E	n_deleted	CRISPResso2
F	n_inserted	CRISPResso2
G	n_mutated	CRISPResso2
H	#Reads	CRISPResso2
I	%Reads	CRISPResso2
J		
K	Edited#Reads	=IF(COUNTIF(A,"*Sequence*"),0,H)
L	Deleted#Reads	=IF(E>0,K,0)
M	Inserted#Reads	=IF(F>0,K,0)
N	Mutated#Reads	=IF(G>0,K,0)
O	DeletedCodon#Reads	=IF(L>0,E,0)
P	Frameshift calling	=IF(INT(O/3)=(O/3),"inframe", "fs")
Q	Frameshift#Reads	=IF(P="fs",H,0)
R	Deletion length	=LEN(A)-LEN(SUBSTITUTE(A,"-",""))

Notes

Sequence references (red) need to be replaced by DNA string for target site

Cell references (blue) need to be followed by row number

Column references (green) need to be specified by a range

Target	Sequence
Bap	TTATCTTCCTGTTCAAATGGATCGAAGAGCGCAGGTCCCGCCGCAAGGTTTCTACGTTGGTGGATGA
Pbrm1	TCCGAGACTATAAGGATGAACAGGGCAGACTCCTCTGTGAGCTGTTCATTAGGGCTCCAAAGCGG
Setd2	AAGGCACGAAGACAAAAGTTAATTTGGAGGAACAGGGACGGCAAA

Input from CRISPResso2

Alleles_frequency_table.txt

Output

Summary	Reads	Frequency
indels:	=SUM(K)	=SUM(K)/SUM(H)
del:	=SUM(L)	=SUM(L)/SUM(H)
fs:	=SUM(Q)	=SUM(Q)/SUM(H)

Table S1. Processing of CRISPResso2 output from amplicon NGS data. The Excel formulas outlined determine the frequencies of on-site edits. In brief, processing of 250 bp paired-end fastq data by the online CRISPResso2 tool generated the “Alleles_frequency_table.txt” file (in which each line (row) represents a unique read and the number of times the exact read was encountered). Upon import into Microsoft Excel, the CRISPResso2 file populated columns A through I of the worksheet outlined above. Columns K-R were populated with row-specific formulas of the formulas specified above (e.g. CELL”L23” has the formula “=IF(E23>0,K23,0)”). Note that the formula in column K contains a target-specific sequence spanning the gRNA target site (or target sites for paired gRNAs). The use of a target-specific search string ensures that SNPs and PCR-introduced mutations outside the target site (denoted by asterisks in Figures S2 and S3) are not erroneously included in the indel counts. Specified above are the paired gRNA target sites for Bap1, Pbrm1, and Setd2. The number of reads for insertions/deletions (indels), deletions (del), and frameshifts (fs), respectively are specified in the output box, and the frequencies of the respective types of genome edits are calculated. The column references (green) are specified by a range (e.g. if the CRISPResso2 output has 10,000 unique reads “=SUM(K)” is specified as “=SUM(K2:K10001)”).

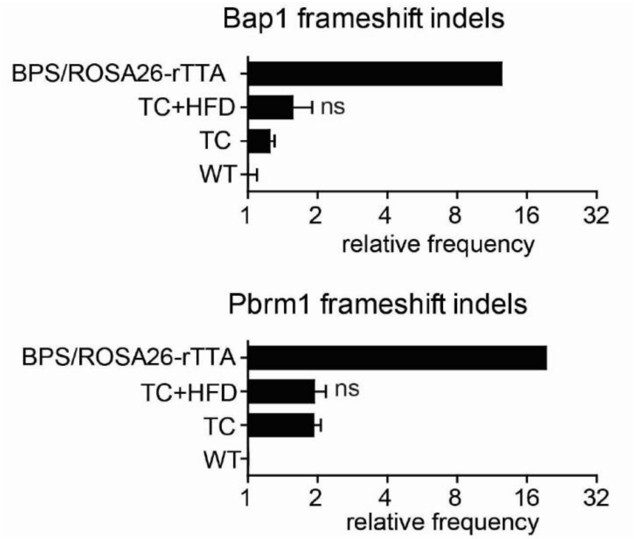

Figure S1. Genome editing of Bap1 and Pbrm1 in BPS-tTA kidneys. To determine the impact on carcinogenesis and mutation frequencies, we sacrificed these mice and examined their kidneys. We detected low frequent frameshift mutations in the kidneys of both dietary groups, but no statistically significant differences in the mutation frequencies.

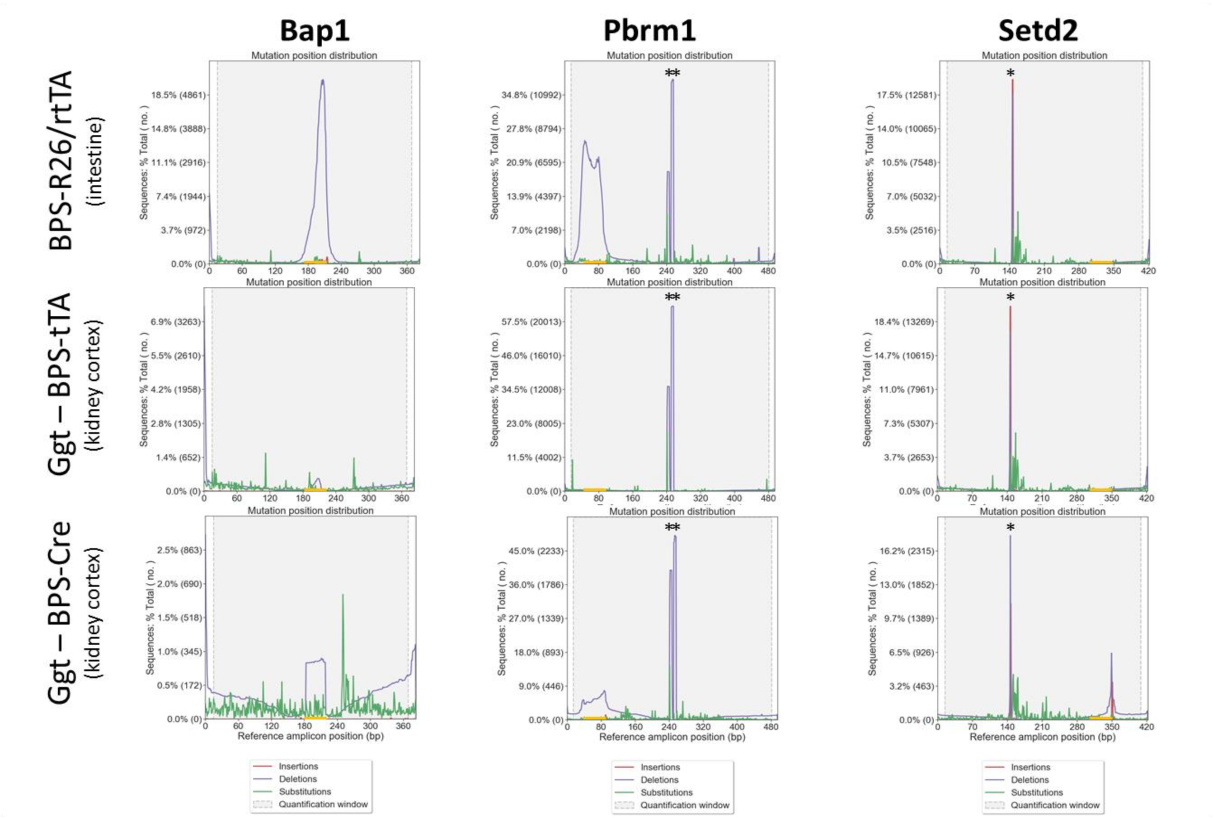

Figure S2. Graphical Representation of Genome Edits in BPS-R26, BPS-tTA, and BPS-Cre mice. The span of the paired gRNA for each target (Bap1, Pbrm1, and Setd2) is indicated in orange. Genomic deletions are denoted in blue. The BPS-R26 line targeted Bap1 and Pbrm1 at high frequencies, whereas almost no editing of Setd2 was observed. The BPS-tTA line targeted Bap1 and Pbrm1 at low frequencies, and no editing of Setd2 was observed. Finally, the BPS-Cre line targeted Setd2 and Pbrm1 at high frequencies, whereas only low frequencies of Bap1 editing were observed. Note that in addition to the targeted region, marked in orange, also genomic variants, marked by asterisks, and truncated PCR products were detected (evident by sloped from termini towards the center). In green, a number of low-frequency substitutions were noted (the irregular locations suggest them to represent PCR artefacts), as well as substitutional hotspots (e.g. positions ~110 and ~270 of Bap1). Insertions in the targeted regions were in most cases observed only at very low frequencies (except for Setd2 targeting in BPS-Cre animals). Depicted are representative editing profiles from one animal from each group. The graphical representations were modified from CRISPResso2 output (4b.Insertion_deletion_substitution_locations.png).

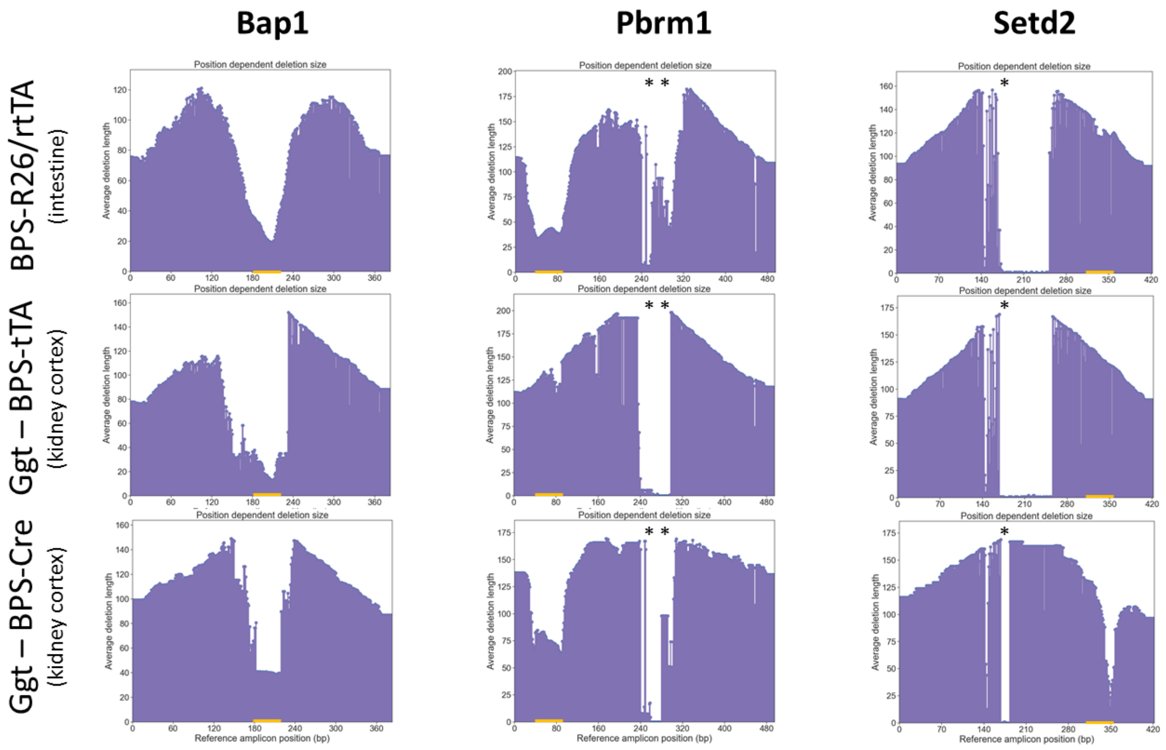

Figure S3. Graphical Representation of Average Deletion Lengths in BPS-R26, BPS-tTA, and BPS-Cre mice. The span of the paired gRNA for each target (Bap1, Pbrm1, and Setd2) is indicated in orange, whereas the sizes of the individual deletions are indicated by lollipops. The valleys in the graphs represent deletions of highly variable sizes (consistent with Cas9-induced non-homology end-joining). Note that for the BPS-Cre animals deletions spanning the entire targeted regions are observed for Bap1 and Pbrm1, whereas the deletions for Setd2 are focally centered on the Setd2-F site (no edits were observed centered on the Setd2-R site). Depicted are representative editing profiles from one animal from each group. The graphical representations were modified from CRISPResso2 output (4d.Position_dependent_average_indel_size.png).

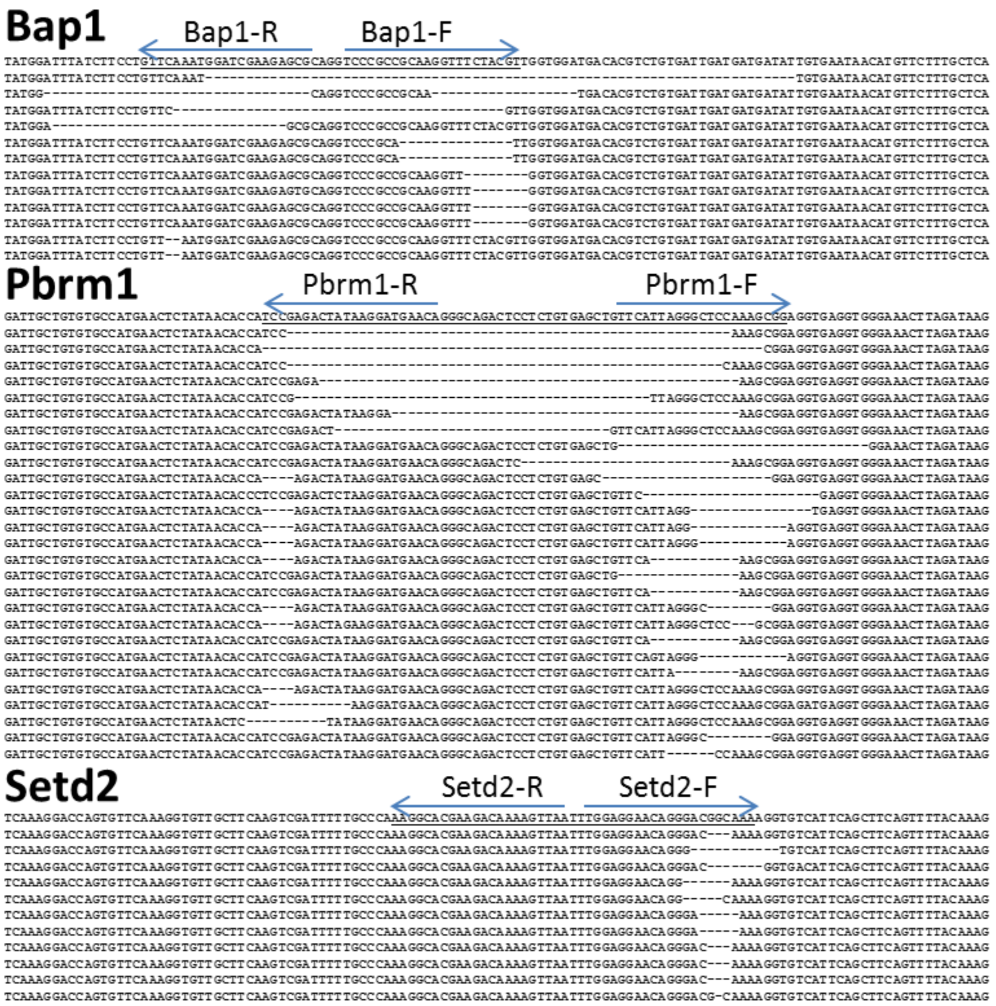

Figure S4. Alignments of Setd2, Bap1, and Pbrm1 Regions from Genome Edited BPS-Cre mice. The span of the paired gRNAs for each target (Bap1, Pbrm1, and Setd2) is underlined. Target sites for individual gRNAs are denoted by blue arrows. Deletions are indicated by hyphens (-). Note that for Bap1 and Pbrm1 the centers of genome editing are located three nucleotides upstream (5'-) of each of the gRNA arrowhead (the break-site), whereas for Setd2 a center of genome editing is observed only for Setd2-F (and not for Setd2-R). Subsets of editing profiles from one BPS-Cre animal are depicted. The alignments were obtained using the online resource CRISPResso2 (Alleles_frequency_table.txt).

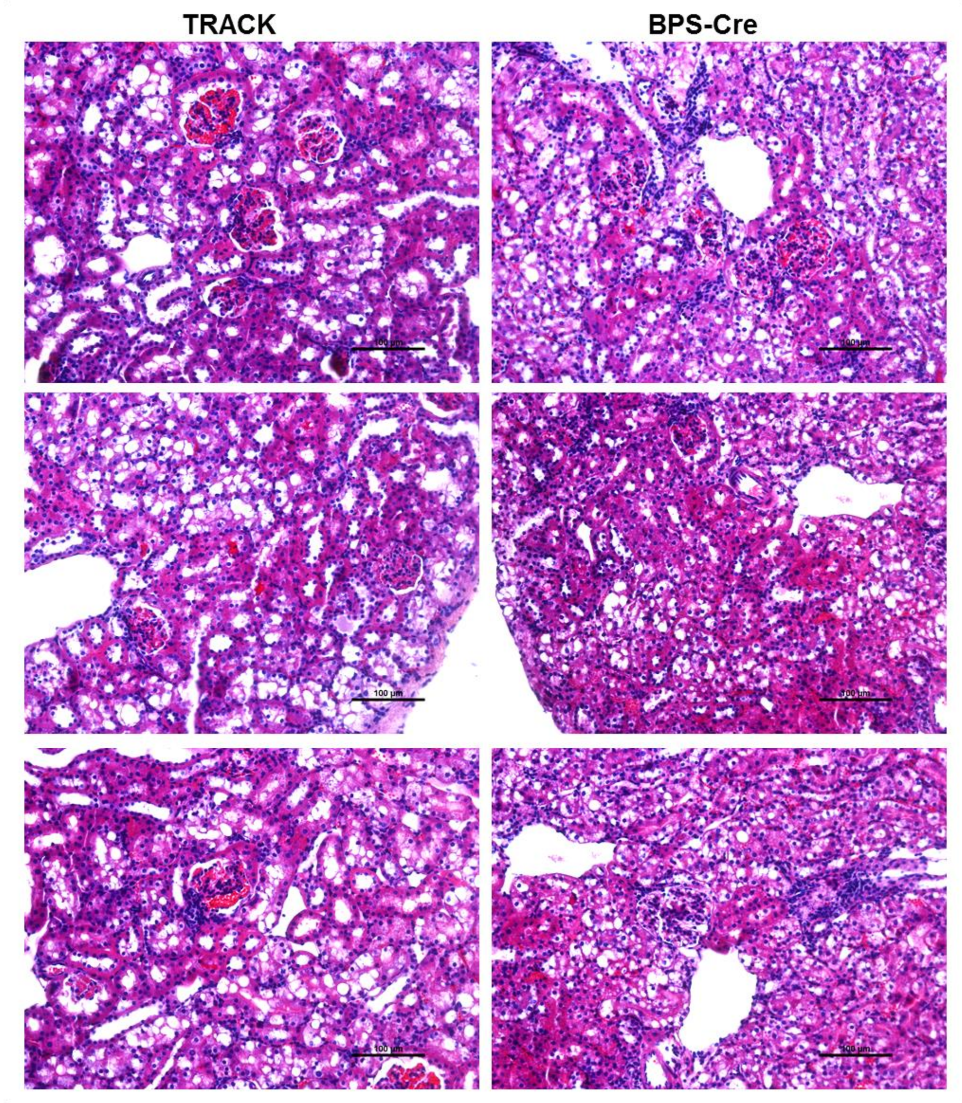

Figure S5. Histological evaluation of BPS-Cre kidneys. The kidneys, as well as various other organs, including livers, lungs and bones, were analyzed for macroscopic abnormalities or signs of malignancy. Despite the observed genome editing, we did not detect macroscopic or microscopic changes in the genome-edited animals, nor did we observe any formation of renal tumor in the BPS-Cre animals. Representative images of kidneys from TRACK and BPS-Cre animals are shown.
